# Prevalence and Risk Factors of Human Herpes Virus Type 8 (HHV-8), Human Immunodeficiency Virus-1 (HIV-1), and Syphilis among Female Sex Workers in Malindi, Kenya

**DOI:** 10.1155/2019/5345161

**Published:** 2019-06-20

**Authors:** M. M. Nzivo, R. M. Lwembe, E. O. Odari, J. M. Kang'ethe, N. L. M. Budambula

**Affiliations:** ^1^Jomo Kenyatta University of Agriculture and Technology, Kenya; ^2^Centre for Virus Research, Kenya Medical Research Institute, Kenya; ^3^Comprehensive Care Centre for HIV, Kenyatta National Hospital, Kenya; ^4^Department of Biological Sciences, University of Embu, Kenya

## Abstract

The prevalence of Human Herpes Virus type 8 (HHV-8), Human Immunodeficiency virus (HIV), and syphilis is high in Sub-Saharan Africa. Studies on HHV-8 in Kenya are few and data on its coinfection with HIV and syphilis scanty. This cross-sectional study among female sex workers (FSWs) in Malindi, Kenya, aimed to determine the prevalence of HHV-8, HIV, and syphilis mono/coinfections and identify associated risk factors. A total of 268 FSWs consented and were administered a structured questionnaire and screened for antibodies against HHV-8, HIV, and syphilis following the National Guidelines. FSWs positive for HHV-8 were 67/268 (25%), HIV 44/268 (16.4%), and 6/268 (2.24%) for syphilis. Eight out of 67 (12%) tested positive for HHV-8/HIV and 2/67 (3%) for HHV-8/syphilis coinfections. Married FSWs had higher odds of HHV-8 infection (OR 2.90, 95%, and* P*=0.043). Single marital status was inversely associated (OR 0.46, 95% CI 0.23-0.94, and* P*=0.034) with HIV infection. HIV was associated with increasing age (OR 14.79,* P*<0.001), inconsistent condom use (OR 2.69,* P*=0.004), increased duration as sex worker ≥6 (OR 3.0,* P=*0.002) and clients ≥4 (OR 4.0,* P<*0.001), intravenous drug use (OR 2.5,* P*=0.043), and early sex debut (*P=*0.049) unlike HHV-8 which was not associated with high risk sexual behavior. HHV-8/HIV coinfection was associated with increasing age (OR 11.21,* P*=0.027). Infection by HHV-8 was not significantly associated with HIV (OR 0.62;* P*=0.257) or syphilis (OR 1.52;* P*=0.636). There was a high likelihood of infection with HHV-8 compared to HIV (OR 8.6,* P*=0.014) and syphilis (OR 14.6,* P*<0.001). The lack of association of HHV-8 with high risk sexual behavior suggests that sexual transmission may not play a significant role in transmission of HHV-8 among FSWs in Malindi.

## 1. Introduction

The prevalence of HHV-8 varies according to geographic region and ethnicity with the highest burden reported in Sub-Saharan Africa (36-60%) and the Amazon where more than half of the population is infected [1, 2]. HHV-8 has been implicated in all forms of Kaposi's sarcoma (KS) including; classic, endemic, iatrogenic and AIDS KS. HHV-8, HIV, and syphilis are transmitted horizontally through sexual, oral, and parenteral route or vertically from mother to child [1, 3, 4]. Studies have reported HHV-8 prevalence to be higher among FSWs than men and women in the general population [5, 6] while others did not record any significant difference [7, 8]. In China prevalence of HHV-8 of between 10 and 16% within the FSW population was reported [8, 9]. In Africa, HHV-8 prevalence of 26% and 20%, 45%, 51.3%, and 44% was reported among FSWs in Djibouti, Nigeria, Cameroon, and Kenya, respectively [10–13].

The HIV burden is disproportionately high among FSWs worldwide. Although the prevalence of HIV has reduced significantly in the general population in Sub-Saharan Africa (6-10%), FSWs still record high prevalence [2, 14]. Whereas the prevalence of HIV in the general population in Kenya in 2016 was recorded at 5.9%, FSWs recorded a prevalence of 29.3%. This was higher than HIV prevalence among men who have sex with men (MSMs) reported at 18.2% and of intravenous drug users (IDUs) recorded at 18.3% [15]. A systematic review consisting West and Central Africa countries reported HIV pooled prevalence of 34.9%, 7.3%, 17.7%, and 3.8% among FSWs, their clients, MSM, and people who inject drugs, respectively [16]. High prevalence of HHV-8/HIV coinfection has been reported in Africa [14, 17, 18] and in Latin America [6]. Syphilis is known to facilitate transmission and acquisition of HIV [19]. Syphilis infection has been associated with HHV-8 by a number of previous studies [13, 20] while others did not report any significant association [7, 8]. Syphilis infection in the general population in Kenya and globally has decreased over years [19, 21, 22]. For instance, in Kenya the prevalence of syphilis in the general female population is approximately 1.7% and estimated to be higher among HIV infected individuals and high risk groups such as FSWs and MSM [19, 22].

The risk factors of these three infections among FSWs have been reported to include inconsistent condom use, increased years of prostitution, high number of sexual partners, and increased sex acts among others [6, 12, 13]. However, there exists a controversy surrounding heterosexual transmission of HHV-8 [3, 7, 8]. HIV and syphilis are of public health concern and HHV-8/HIV coinfection has been associated with Kaposi's sarcoma the leading AIDS defining illness in Kenya. Female sex workers act as a bridge for transmission between this population and the general low risk population. With the rampant sex tourism and international travel to trade, a high rate of spread of HHV-8, HIV, and syphilis from regions of high prevalence to those of low prevalence such as Europe and North America is anticipated; thus they may not remain restricted but could spread globally. Therefore, understanding prevalence and risk factors associated with HHV-8/HIV/syphilis mono- and coinfections remain a prerequisite for development of strategies for prevention of these infections. The results of this study give insight on the prevalence and risk factors of HHV-8, HIV, and syphilis and their coinfections within the high risk FSWs population.

## 2. Methodology

### 2.1. Study Area

A cross-sectional study was carried out among FSWs in Malindi, in the coastal region of Kenya. Economically, the region relies heavily on tourism and attracts people from different parts of the world and country. The region is a cosmopolitan with different religions, cultures, and races. Many FSWs from within and outside the region flock to this town and engage with tourists in sex tourism. Tourists frequenting Malindi are mainly from Europe, with tourists from Italy and Germany accounting for the highest proportion. Women who were ≥18 years and reported to be exchanging sex for money to men were recruited. They were recruited into the study through two drop-in centres; the Ananda Marga Universal Relief team (AMURT) Health Centre and the Kenya NGO AIDS Consortium (KANCO) Drop-In Centre. The centres offer services mainly to FSWs and MSMS.

### 2.2. Ethical Approval

This study was authorized by the independent Scientific and Ethics Review Unit (SERU) of the Kenya Medical Research institute (KEMRI) (KEMRI/CVR/SERU NUMBER 2915). This study was also authorized by the Department of Health, Kilifi County. All ethical considerations regarding the use of human subjects were followed.

### 2.3. Sampling and Collection of Data on Sociodemographics and Behavioral Characteristics

Purposive random sampling was carried out. Females visiting the centre were approached by the PI and enquired about their purpose for visit and if they were FSWs. Upon confirming that they were sex workers, they consented and were recruited. FSWs visited AMURT Health Centre for either treatment or family planning procedures, to collect condoms or to attend routine meetings among others. In KANCO Drop-In Centre, FSWs visited to collect injecting needles, for treatment and counseling meetings among others. New recruits to the centres who were brought in the course of the study by peer educators were also included. A structured questionnaire was administered by a health worker to all the participants to collect data on sociodemographic characteristics including age, level of education, and marital status. Information on medical history including HIV status and history of STIs was also recorded. Other information recorded was on sexual behavior characteristics specifically the number of years as a sex worker, age at first sexual intercourse, intravenous drug use, number of clients per day, and use of condom.

### 2.4. Sample Collection, Screening, and Laboratory Testing

After consenting and the questionnaire information was captured, 5 millilitres of venous whole blood was obtained from each study participant by a trained and certified phlebotomist. The whole blood separated into cells and plasma. Plasma was used for serological testing of HHV-8, HIV, and Syphilis. HHV-8 Enzyme Linked Immunosorbent Assay (Sunlong Biotechnologies Ltd) was used to detect HHV-8 igG antibodies in plasma samples. This utilized the sandwich ELISA technique. Using a micro Elisa strip plate, 50 *μ*l of both negative control and positive control were added in two wells each and one well was left empty as a blank control. In sample wells, 40 *μ*l of sample dilution and 10 *μ*l of each sample were added. The plate was sealed and incubated for 30 min at 37°C. The seal was removed and washed 5 times. In all wells except the blank, 50 *μ*l of HRP-conjugate reagent was added. The plate was incubated and samples washed again as described earlier. A stop solution (50 *μ*l) was then added to terminate the reaction. There was a color change from blue to yellow in some wells. The absorbance O.D was then read at 450 nm using a micro titre plate reader. A cutoff value was calculated by averaging the value of two negative controls plus (+) the value 0.15 as directed by the manufacturer Positive samples had an O.D above the cut off value.

Detection of HIV in plasma samples was done following the National Algorithm for HIV testing. A parallel HIV testing algorithm using plasma samples was done with Determine™ (Alere Medical Co. Ltd) and First Response® HIV 1-2-0 (Premier Medical Co. Ltd) following manufacturer's instructions. A sample was deemed positive if it reacted with both kits. Indeterminate results were confirmed using ELISA kit murex.

Detection of syphilis was done using a one-step Anti-TP test and confirmed with* Treponema pallidum *hemagglutination assay using plasma samples.

### 2.5. Statistical Analysis

Field and laboratory data were entered in SPSS version 22 where frequencies were calculated to determine the prevalence of HHV-8, HIV, and syphilis. Social demographic characteristics and risk behaviors were analyzed using descriptive statistics, that is, mean, median, and range for age and frequencies for categorical variables; age groups, marital status, level of education, HIV, syphilis, and HHV-8 positivity, condom use, number of clients, number of years as a sex worker, and the age one became sexually active. All data was also converted to binary form in excel and then subsequently transferred to Stata version 10 where odds ratios (OR), 95% confidence intervals (CI), and p values were estimated by logistic regression.* P*<0.05 was considered statistically significant.

## 3. Results

### 3.1. Basic Characteristics of the Study Participants

The study recruited 268 FSWs with a median age of 28. A majority 87 (32.5%) of FSWs were in age groups 25-29, 74 (27.6%) 18-24, 55 (20.5%) 30-34, and 52 (19.4%) above 35 years. Single FSWs were 180 (67.2%) and the widowed/separated 62 (23.1%), while the married were 26 (9.7%). Majority FSWs had primary level of education 151 (67.2%) and 85 (31.7%). Only 26 (9.7%) FSWs were IDUs. Most FSWs had 1-3 clients per day 139 (51.9%) while 129 (48.1%) had ≥4. Majority used condom consistently 195 (72.8%) while 73 used it (27.2%) occasionally. Sixty-eight (25.4) FSWs had been in the business for ≥6 years while 200 (74.6%) for 1-5 years. Up to 237 (88.4%), FSWs started engaging in sexual intercourse while they were <18 years of age while the rest 31 (11.6%) ≥18 years. Thirty-nine (39) of the 44 participants who were positive for HIV already knew their HIV serostatus and were on treatment, either on ART or cotrimoxazole therapy (Table 1).

### 3.2. Prevalence of HHV-8, HIV, and Syphilis Infections and Coinfections among FSWs

**Figure 1:**
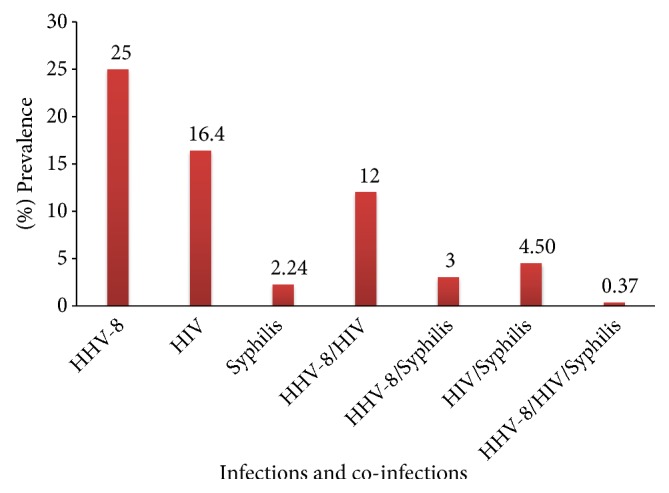
The prevalence of HHV-8, HIV and syphilis mono and co-infections among female sex workers in Malindi, Kenya in 2015-2016.

In this study, 67/268 (25%) FSWs samples tested positive for HHV-8, 44/268 (16.4%) for HIV, and 6/268 (2.24%) for syphilis. FSWs tested positive for HHV-8 /HIV and HHV/syphilis and HIV/ syphilis were 8/67 (12%), 2/67 (3%), and 2/44, respectively. Only one out of 268 (0.37%) FSWs samples tested positive for HHV-8, HIV, and syphilis (Figure 1).

### 3.3. Likelihood of Infections

The prevalence of HHV-8 (25%) was significantly different from HIV prevalence (16.4%)* P*=0.0143 and syphilis prevalence (2.24%)* P*<0.001. HIV prevalence was significantly different from syphilis prevalence* P*<0.001. The prevalence of coinfections was not significantly different. The prevalence of HIV/syphilis coinfection was not significantly different from HHV- 8/HIV* P*=0.19 and HHV-8/syphilis prevalence* P*=0.7 (Table 2).

### 3.4. Risk Factors Associated with HHV-8, HIV, and Syphilis Infections among FSWs

There was a significant association between HHV-8 infection and being married (OR 2.90;* P*=0.042). An inverse association between HIV infection and single marital status OR 0.46 (CI 0.23-0.94) was observed. HIV was significantly associated with increasing age: ≥35 years age (OR14.79;* P*≤0.001), 30-34 (4.63;* P*=0.027), and 25-29 (OR 3.78;* P*=0.046). HIV was associated with all high risk sexual behavior unlike HHV-8. There was a significant association between HIV (OR 2.5;* P*=0.043) and IDU contrary to HHV-8 (OR 0.89;* P*=0.812) and HIV infection (OR 4.0;* P*<0.001) with increased number of clients ≥4 clients unlike HHV-8 (OR 0.9;* P*=0.724). Inconsistent condom use was significantly associated with HIV infection (OR 2.69;* P*=0.004) but not with HHV-8 (OR 0.98;* P*=0.937) similar to increased number of years as a sex worker ≥6, HIV infection (OR 3.0;* P*=0.002), and HHV-8 infection (OR 0.639; p=0.197). Early sex debut <18 was also weakly associated with HIV infection (OR 0.42;* P*=0.049). Syphilis infection was not associated with any of the evaluated socio-demographic or sexual behavior characteristics. However, the number of FSWs who were infected by syphilis was few and infection only occurred in two age groups: 25-29 and above ≥35 years (Table 3).

### 3.5. HHV-8/HIV and HHV-8/Syphilis Coinfection

HHV-8/HIV coinfection was associated with increased age (≥35, OR 11.21;* P*=0.027). FSWs who were IDUs (OR 3.2;* P*=0.16), who had ≥4 clients in a day (OR 3.3;* P*=0.144) and who inconsistently used condom (OR 1.63;* P*=0.512), were all at an increased risk of HHV-8/HIV infection but there was no significant association. In case of HHV-8/syphilis coinfection, FSWs ≥35 years (OR 1.68;* P*=0.714) and who inconsistently used condom (OR; 2.69;* P*=0.485) recorded higher odds of infection but no significant association with HHV-8/syphilis coinfection was observed.

### 3.6. HHV-8, HIV and Syphilis Mono/Coinfections with Age

**Figure 2:**
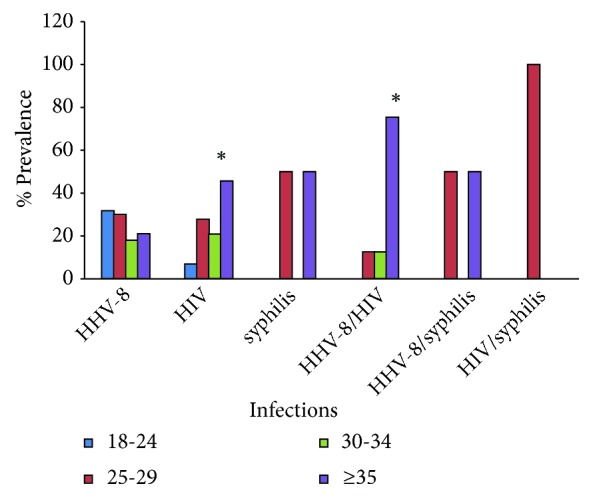
The prevalence of HHV-8, HIV, and syphilis mono- and coinfections across different age groups among female sex workers in Malindi, Kenya, in 2015-2016. Asterisks (*∗*) show infections that recorded significant association with age.

HHV-8 infection was recorded at 31%, 30%, 21%, and 18% in age groups 18-24, 25-29, ≥35, and 30-34, respectively. HHV-8 infection was not significantly associated with age (*P*=0.792). HIV infection in different age groups: 46% (≥35), 7% (18-24), 26.5% (25-29), and 20.5% (30-34). HIV was significantly associated with age (*P*<0.001). Syphilis infection was observed in the age groups 25-29 and ≥35 only. HHV-8/HIV coinfection increased from 0% from age group 18-24 to 75% among FSWs aged ≥35 years (*P*<0.001). HIV/syphilis coinfection was only recorded at age group 25-29. HHV-8/syphilis coinfection was recorded at 50% in age groups 25-29 and ≥35 (Figure 2).

## 4. Discussion

This study reported HHV-8 prevalence of 25% among FSWs in Malindi, Kenya. This is lower than HHV-8 prevalence from previous studies in Mombasa, Kenya, among HIV-1-seronegative prostitutes and drivers of a trucking company which were reported as 44% and 43%, respectively [13, 23]. This could be as a result of improvement of living standards as HHV-8 in Sub-Saharan Africa has largely been reported to be transmitted by non-sexual route. HHV-8 infection was significantly associated with being married. The reason for this could not be explained within this study. HHV-8 association with being married has also being reported [7, 24, 25] but other studies noted there was no association [8, 26, 27]. HHV-8 was not associated with age. HHV-8 was at peak among women FSWs 18-24 years of age indicating that it may have been acquired earlier in life. Lack of association of HHV-8 with age has been observed by other studies [7, 10, 12, 28, 29] but was inconsistent with other studies which recorded a significant association [5, 13, 26].

The prevalence of HIV (16.4%) in this study was higher than in the general population (5.9%) in Kenya but lower than the recorded overall prevalence of HIV among FSWs (29.3%) in Kenya [15]. Studies among FSWs in Nairobi and Kisumu reported HIV prevalence of 29.5% and 56.5%, respectively [19, 30]. Studies among FSWs in Rwanda, South Africa, and Central Africa recorded HIV prevalence of 42.9%,76.8%,and 6.3-39.1%, respectively [7, 31, 32]. Low HIV prevalence was recorded among FSWS in Asia (0-5.6%) and in the Middle East and Northern African Region 4% [33–36]. The lower HIV prevalence in this study compared to overall prevalence of FSWs in Kenya could be as result of structural interventions aimed to curb HIV in this population including creating awareness, frequent testing, supplying condoms, and campaigns against sexual violence and provision of needles and syringes to IDUs to avoid sharing among others. HIV infection was associated with age similar to other studies among FSWs [19, 32]. This could be as a result of continuous exposure over years. HIV was also associated with high risk sexual behavior such as inconsistent condom use, increased number of years as sex worker, number of clients, and intravenous drug use similar to other studies [32, 34, 36]. An inverse association of HIV infection with single marital status was observed similar to study in Mombasa, Kenya [37]. This could be because the latter are younger and more informed about HIV and have found established measures aimed at curbing HIV.

Syphilis infection was 2.24% higher than the recorded prevalence in the general female population in Kenya (1.7%) and among FSWs in Nairobi reported at 0.9% [19]. High syphilis prevalence was recorded among sex workers in Rwanda 51.1% and in South Africa 19% [7, 32]. Studies among FSWs in Iran, India, and China recorded syphilis prevalence of 0%, 3.6%, 6.9%, and 10.5%, respectively [8, 34–36]. Low syphilis prevalence in this study could be attributed to enhanced use of condoms and frequent testing through targeted programs for FSWs. In addition syphilis has clear symptoms and, with availability of health care services, one can be easily treated using antibiotics upon infection. However, detection of syphilis was also a clear sign that preventive measures such as condom use needed to be pursued more.

The prevalence of HHV-8/HIV coinfection (12%) was lower than prevalence of coinfection among prostitutes in Jos Nigeria and Imbituba Brazil reported as 20% and 16.4%, respectively [6, 12]. HHV-8/HIV coinfection was significantly associated with age. This could be as a result of increased HIV infections due to continuous exposure over years and reactivation of HHV-8 hence seroconversion due to a weakened immune system or new infections. HHV-8/syphilis coinfection observed in the study was minimal (3%). A HIV/syphilis coinfection recorded of 4.5% was lower than 27.4% recorded among FSWs in Rwanda [32]. Coinfections were an indicator to unprotected sexual behavior within this group.

In the present study HHV-8 infection was not associated with HIV or syphilis infection. This could be due to the fact that HHV-8 and HIV presented with different modes of transmission in this population that is sexual and non-sexual. This has been reported by other studies [7, 8, 23, 25, 26, 28] but contrasted with some which reported significant association [12, 27, 38]. HHV-8 infection did not present with characteristics of a STI. This is because unlike HIV infection, HHV-8 was not associated with high risk sexual behavior. However, transmission of HHV-8 earlier in life could have masked sexual transmission of HHV-8 if it did occur. Nonsexual transmission of HHV-8 may be the predominant route through which HHV-8 is transmitted in Kenya. A review from studies on HHV-8 among FSWs around the world observed that high risk behavior and STIs were not associated with HHV-8 infection by majority of studies evaluated; concluding FSWs did not play a major role in HHV-8 transmission hence heterosexual transmission [39]. We interpret our results as indicating that sexual transmission of HHV-8 occurs rarely in this population.

### 4.1. Conclusion

There is a high prevalence of HHV-8 and HIV among FSWs in Malindi, along the Kenyan coast. Although there are ongoing measures to curb HIV in this population, much more need to be done. There is a need to create awareness on HHV-8. To the best of our knowledge this is the only study in the country providing data on prevalence of HHV-8 monoinfection, HHV-8/HIV, and HHV-8/syphilis coinfection among both HIV negative and positive FSWs.

### 4.2. Limitations

As this was a cross-sectional study, it was impossible to determine behavior at time of seroconversion. Further, this study was conducted within a group that had been enlightened on the dangers of sex work and is trained to live positively and hence may not give a true picture of FSWs in Kenya. We finally note that the participants were mainly drawn from harm reduction centres, potentially giving room for inherent biases including selection bias. Such biases may have contributed to under- or overreporting of the true status of the study population.

## Figures and Tables

**Table 1:** Basic characteristics for female sex workers in Malindi, Kenya in 2015-2016.

Characteristic	Number	Percentage (%)
*Age*		
35 and above	74	27.6
30-34	87	32.5
25-29	55	20.5
18-24	52	19.4
*Marital status*		
Single	180	67.2
Married	26	9.7
Widowed/separated	62	23.1
*Education Level*		
Primary	151	56.4
Secondary	85	31.7
Tertiary*∗∗*	2*∗∗*	0.7
NonFormal	30	11.2
*IDU*		
Yes	26	9.7
No	242	90.3
*Clients per day*		
4 and above	129	48.1
1 to 3	139	51.8
*Condom use*		
Occasionally	73	27.2
Consistently	195	72.8
*Duration as a sex worker in years*		
≥6	68	25.4
1-5	200	74.6
*Sex debut*		
<18	237	88.4
≥18	31	11.6

IDU-intravenous drug users, (*∗∗* number too small to draw statistical inference).

**Table 2:** Likelihood of infections.

Condition	n(N)	Prevalence	OR	p value
*Mono infections*				
HHV-8	67(268)	25	14.6	0.001
HIV	44(268)	16	8.6	0.001
Syphilis	6(268)	2.2	Reference	
*Co-infections*				
HHV-8/HIV	8(67)	11.9	2.8	0.19
HHV-8/Syphilis	2(67)	3	0.6	0.7
HIV/Syphilis	2(44)	4.5	Reference	

**Table 3:** Percentage of HHV-8, HIV and syphilis positivity, OR, 95%Cl and p-values of risk factors associated with HHV-8, HIV and syphilis positivity among female sex workers in Malindi, Kenya in 2015-2016.

Risk factors	268	HHV-8 Infection		P value	HIV Infection		P value	Syphilis Infection		P value
		n (n/67%)	OR (95% CI)		n (n/44%)	OR (95% CI)		n (n/6%)	OR (95% CI)	
*Age*										
35 and above	52	21 (31.3)	0.92 (0.42-2.06)	0.858	20 (45.4)	14.79 (4.10-53.37)	*0.001*	3 (50)	1.7 (0.33-8.23)	0.519
30-34	55	20 (29.9)	0.70 (0.31-1.59)	0.399	9 (20.5)	4.63 (1.19-18.01)	*0.027*	0	1	
25-29	87	12 (17.9)	0.75 (0.37-1.53)	0.435	12(27.3)	3.78 (1.02-13.97)	*0.046*	3(50)	1	
18-24	74	14(20.9)	1		3 (6.8)	1		0	1	
*Marital status*										
Single	180	46 (68.7)	1.59 (0.76-3.31)	0.214	25 (56.8)	0.46(0.23-0.94)	*0.034*	4 (60)	0.68 (0.12-3.82)	0.663
Married	26	10 (14.9)	2.90 (1.04-8.07)	*0.042*	3 (6.8)	0.38 (0.99-1.41)	0.149	0	1	
Widowed/separated	62	11 (16.4)	1		16 (36.4)	1		2 (40)	1	
*Education Level*										
Primary	151	37 (55.2)	0.89 (0.37-2.17)	0.802	20 (45.5)	0.99 (0.31-3.14)	0.99	3 (50)	0.58(0.05-5.85)	0.65
Secondary	85	21 (31.3)	0.90 (0.35-2.33)	0.832	19 (43.2)	1.87 (0.58-6.03)	0.294	2 (33.3)	0.70 (0.06-8.00)	0.773
Tertiary*∗∗*	2*∗∗*	1(1.5)	2.75 (0.2-49.36)	0.492	1 (2.3)	6.5(0.34- 126.06)	0.216	0	1	
NonFormal	30	8 (11.9)	1		4 (9.1)	1		1 (16.7)	1	
*IDU*										
Yes	26	6 (9.0)	0.89 (0.34 -2.31)	0.812	8 (18.2)	2.5 (1.03-6.29)	*0.043*	0	omitted	
No	242	61 (91.0)	1		36 (81.8)	1		6(100)		
*Clients per day*										
4 and above	129	31(46.3)	0.9 (0.52-1.57)	0.724	33(75)	4.0 (1.92-8.32)	*0.001*	3 (50)	1.08 (0.21-5.45)	0.926
1 to 3	139	36 (53.7)	1		11(25)	1		3(50)		
*Condom use*										
Occasionally	73	18 (26.9)	0.98 (0.52-1.82)	0.937	20 (45.5)	2.69 (1.38-5.25)	*0.004*	5 (83.3)	1.3 (0.31-5.74)	0.556
Consistently	195	49 (73.1)	1		24 (54.5)	1		1 (16.7)	1	
*Duration as a sex worker in years*										
≥6	68	13 (19.4)	0.63 (0.32-1.26)	0.197	18 (40.9)	3.0 (1.51-6.02)	*0.002*	2 (66.7)	1.5 (0.34-6.34)	0.596
1-5	200	54 (80.6)	1		26 (59.1)	1		4 (33.3)	1	
*Sex debut*										
< 18	237	59 (88.1)	0.95 (0.40-2.24)	0.912	35 (79.5)	0.42(0.18-1.00)	*0.049*		0.44 (0.08-2.27)	0.329
≥18	31	8 (11.9)	1		9 (20.5)	1			1	

IDU-intravenous drug users, (*∗∗* number too small to draw statistical inference), Italicized numbers show where significant association was observed.

## Data Availability

The SPSS and STATA files with the data used to support the findings of this study are available from the corresponding author.
